# The importance of margins status after breast conservative surgery and radiotherapy in node positive patients: a follow-up of 10–15 years

**DOI:** 10.1186/1477-7800-5-13

**Published:** 2008-05-22

**Authors:** Isabella Besana-Ciani, Michael J Greenall

**Affiliations:** 1 Department of Surgery, Oxford Radcliffe NHS Trust, Oxford, UK; 2 Level 2 Medical Secretariat, John Radcliffe Hospital, Headley Way, Headington, Oxford, OX3 9DU, UK

## Abstract

**Background:**

Margin status is the main factor determining local recurrence (LR) after wide excision and radiotherapy for breast cancer. The aim of the study is to evaluate if positive margins are as great a risk factor for LR in node-positive as in node-negative patients, since the major risk in the former group is dissemination and whether there is a correlation between nodal status and margins in relation to prognosis.

**Methods:**

773 patients underwent WLE and radiotherapy between 1988 and 1992 and were followed-up (> 10 years) to determine LR rates according to margin and nodal status. Margins were assessed by cavity-shave biopsies and the axilla was staged by sampling or clearance.

**Results:**

461 patients were node negative and 312 node positive. In the node-negative group 415 patients had negative margins and 46 positive: LR after > 10 years was 12 % and 28 % respectively. Among the 312 patients in the node positive group, 267 were margin negative and 45 positive; the LR rate was 12 % and 18 % respectively. In the node negative-group there was a statistically significant difference between the positive and the negative margins with higher relapse rate and lower overall survival (p < 0.001), whereas in the node-positive group the equivalent comparison didn't show any statistical difference.

**Conclusion:**

Although re-excision should be always recommended, in node-negative patients positive margins are associated with a statistically higher LR rate and lower overall survival while in node-positive disease margins might be of less importance in determining prognosis as dissemination is more likely to occur.

## Background

Breast conservation with adjuvant radiotherapy is standard therapy for early stage invasive breast cancer. Randomized trials comparing breast conservation with mastectomy have consistently demonstrated similar survival in the two groups [1,2] but with increased local recurrence rates in patients undergoing conservative surgery, especially if radiotherapy is omitted [3]. Local recurrence has been seen as one of the major drawbacks of breast conservation although there had been a previous belief that salvage mastectomy in such circumstances would not necessarily be associated with any survival disadvantage [1]. Unfortunately it became apparent that up to 50% of patients with local recurrence have inoperable disease and that local recurrence is often associated with dissemination [4]. As a result attempts have been made to minimize local recurrence and to better define both those factors which predispose to its development. Despite this many issues remain unresolved.

The most important factor leading to local recurrence after breast conservation and adjuvant radiotherapy, and the only one under surgical control, is the presence of positive margins at the limit of excision. However, there is little consensus on either how to assess or quantify extent of clearance following such surgery [5]. Other important factors predisposing to local recurrence include the presence of extensive in-situ change, poor tumor grade, widespread lymphovascular invasion and possibly young age at presentation of disease. Less significant, although of some importance, are factors such as tumor size, histological type, nodal involvement and provision of adjuvant drug treatment [5,6].

As there has been increasing evidence that local recurrence might be associated with enhanced risk of metastatic disease and worsening prognosis, surgeons should attempt to minimize local recurrence when performing breast conservation by virtue of good case selection and ensuring negative margins after surgical excision [4,7].

The aim of the current study was therefore to determine if positive margins following wide excision and adjuvant radiotherapy are as great a risk factor for local recurrence in node positive as in node negative patients, in the belief that the major risk in this group was dissemination rather than local recurrence.

It would also evaluate whether there is correlation between nodal status and margin positivity in relation to prognosis and overall outcome, to determine how these results apply as guide on re-excision policy.

## Methods

### Patients

Seven hundred and seventy three women with stage I/II invasive ductal carcinoma of the breast were treated consecutively between 1988 and 1992 by the same surgeon in a single unit at the Department of Surgery, John Radcliffe Hospital, in Oxford. In all cases the tumor was the first primary cancer and all patients underwent breast conservative surgery with axillary staging followed by post operative radiotherapy using tangential fields, including an additional boost to the operative field.

### Surgical and adjuvant treatment

Margins of excision were assessed using cavity shaves, if these demonstrated no evidence of either in-situ or invasive disease excision was assumed to be complete. Positive margins were defined by the presence of either in situ or invasive disease within one or more of the four separate margin specimens. Involved margins were not routinely re-excised, according to the policy adopted in Oxford before 1992, which was based on the belief that radiotherapy would have negated the effect of eventual residual disease. This policy allowed us to evaluate the outcome of this group of non-reexcised positive margins and to compare it with the group in which complete excision was achieved.

Axillary nodal status was assessed by either clearance or sampling; those patients with node involvement had axillary irradiation if block dissection was not performed.

Adjuvant treatment involved chemotherapy with CMF for premenopausal patients and Tamoxifen for postmenopausal patients, according to the protocol adopted at that time.

### Follow-up

All patients were followed up prospectively by the Clinical oncology department of Cancer Research UK and entered on a computerized data base which is updated annually. All patients with local recurrence from the cohort under review were identified from the database with a follow-up of at least 10 years. Follow-up time was calculated from the date of surgery to the date of last database update or death. Median follow-up time was 132 months (range 16–182). Only those with local recurrence in the treated breast were included in the analysis; patients who developed disease in the contralateral breast were excluded.

Patients have been divided in two main groups, according to their nodal status and for each group local recurrence rates and overall survival have been analyzed comparing patients with positive margins and those with negative margins after wide local excision and adjuvant radiotherapy [Table 1]

**Table 1:** Node negative group

	Margins -ve	Margins +ve	P value
N (461)	415	46	
LR (at 10 years)	50 (12%)	13 (28%)	p < 0.001 *
10 years survival	83%	61%	p < 0.001 *
			*statistically significant

N: number of patients

LR: local recurrence

### Statistical Evaluation

In all four groups overall survival was calculated using the method of Kaplan – Meier. Comparative survival analysis and statistical comparison of local recurrence rates was performed using Pearson Chi-Square test and Fisher exact test.

## Results

### Local Recurrence

Seven hundred and seventy three patients were analyzed, 461 were node negative and 312 were node positive.

In the node negative group there were 415 patients with negative margins and 46 with positive margins; the local recurrence rate, after a minimum of 10 years, was 12 % and 28 % respectively [Table 1]. This difference was statistically significant (p = < 0.001, Chi Square Test). Furthermore, 10 yr survival in patients with negative margins was 83% compared to 61% in those with positive margins (p = < 0.001, Chi Square Test). Among the 312 patients in the node positive group, 267 had negative margins and 45 had positive margins after primary surgery. Local recurrence rate was 12% and 18 % respectively [Table 2]. This difference did not achieve statistical significance (p = 0.13, Chi-square Test). Ten year survival in the node positive group was 58% in those with negative margins and 47% in patients with positive margins (p = 0.14, Chi Square Test).

**Table 2:** Node positive group

	Margins -ve	Margins +ve	P value
N (312)	267	45	
LR (at 10 years)	33 (12%)	8 (18%)	p < 0.13
10 years survival	58 %	47 %	P < 0.14

N: number of patients

LR: local recurrence

### Survival

Distant relapse rates and overall survival at ten years have also been examined for each group. In the node negative group, patients with negative margins had a 20% distant relapse rate while patients with positive margins had a 26% rate; overall survival at 10 years for node negative patients was respectively 83% in the negative margins group and 61% in the positive margins group, leading to a relevant statistical difference between the two (p < 0.0001) in terms of outcome.

In the node positive group, as expected, rates of distant relapse were significantly higher compared to the node negative group, 41% and 49% respectively, but similar between patients with negative and positive margins.

Consequently also overall survival at 10 years was evidently lower, resulting in a 58 % for the negative margins group and 47 % for the positive margins group; again in this node positive group the comparison between the subgroups according to margins status was not statistically significant (p < 0.14).

## Discussion

### Margin status and local recurrence

Factors associated with local recurrence after breast conservation surgery with adjuvant radiotherapy include completeness of excision, extensive intraductal component, lymphovascular invasion, grade and young age. Less important are tumour size, nodal involvement and histologic type.

Most significant is margin status and is the only factor under surgical control [5,6]. There is little consensus regarding evaluation of margin status with variations in opinion concerning whether inked margins or cavity shaves be used to assess completeness of excision and how extensive that clearance should be. Previous suggestions [5] that a 2 cm margin of clearance was necessary have now been been modified such that a little as 2 mm is now recommended by some authorities [6,8]. This is because of the increasing realization that up to 40% of patients demonstrate multifocality more than 2 cm from the margin of the tumour and that even with extensive clearance cancer or pre-cancerous change is likely to remain in the breast [4,7]. If radiotherapy is omitted after breast conservation surgery the local recurrence rate is up to 30% even in prognostically favorable cases because of the deleterious effect of this multifocal disease [3,9].

Leaving positive margins in-situ is the single biggest risk factor for local recurrence. Radiotherapy and systemic drug treatment may limit such recurrent disease but cannot reduce it to levels observed in patients with negative margins [5,10]. The likelihood of recurrence in patients with positive margins is dependant on the definition of positivity, the extent of disease, the duration of follow-up and whether in-situ or invasive cancer remains. Quantification of the factors is difficult: some centers describe margin status from examination of inked margins of the main specimen whereas others prefer the use of 'cavity shavings'. Comparisons show little difference between these two techniques although cavity shaving is not widely used outside the UK [11].

### Local recurrence and prognosis

Although local recurrence rates up to 40% have been described in the literature [8] good surgical technique, appropriate case selection and routine post-operative radiotherapy should result in a much lower incidence of such disease. Indeed, guidelines from the 'British Association of Surgical Oncology' indicate a target of 5% local recurrence rate at five years [12].

Local recurrence was previously regarded as having little prognostic importance. Salvage mastectomy after local recurrence may be associated with 5-year survival rates up to 84% which is little different to that in patients without local recurrence. The additional belief that any potentially harmful effects of leaving positive margins in-situ could be abrogated by radiotherapy previously led to a less aggressive approach in achieving margin negativity. More recent data, however, indicates a clear relation between local recurrence and risk if distant relapse [4,13]. The NSABP study showed the risk of distant relapse was 3.41 times greater after correction for other factors such as tumour size and type and that it was a strong independent predictor of metastatic disease [13]. These authors emphasized, however, that local recurrence was a marker and not necessarily a cause of distance relapse. Veronesi et al claimed patients under 35 years of age at presentation who locally relapsed within two years of diagnosis were particularly at risk from metastatic disease [4]. A more recent review on the subject has again provided more evidence of the relationship between local recurrence and risk of metastatic disease and subsequent reduction of survival [14]. These findings have resulted in a much more aggressive approach to margin negativity after breast conservation surgery such that re-excision or mastectomy is now regarded as essential in all patients demonstrating incomplete removal of their cancer at the time of their initial operation.

### Nodal status

Survival of breast cancer is particularly dependent on nodal status, tumour size, grade and ER status. Nodal involvement is the most important predictor of systemic relapse and is itself determined by the number of nodes involved, the level of involvement in the axilla and perhaps the size of metastases. Advocates of routine block dissection have always claimed that this is the only way to accurately assess the axilla albeit at the expense of increased arm morbidity and the knowledge that up to 70% of patients are node negative and will therefore be overtreated by this technique. The sensitivity and specificity of sentinel node biopsy is without doubt although questions remain as to the clinical importance of minor degrees of metastatic spread identified by immunohistochemical evaluation of such nodal tissue. Nodal sampling, used predominately in the UK, has been criticized because of its potential qualitative inaccuracies although those studies comparing it to block dissection or sentinel node biopsy have shown few differences in axillary relapse rates or survival. While survival is undoubtedly related to the axillary tumor burden at presentation most important prognostic information is gained from the grouping of nodal positivity scores. For practical purposes knowledge of overall outcome is based on whether 1–3, 4–9 or more than 9 nodes are involved rather than from a precise nodal count because of the effect of other prognostic factors such as tumour size or grade.

### The competitive effect between margins and nodal status

The likelihood of local recurrence is therefore a competitive effect between those factors leading to local recurrence and those predicting systemic relapse. In our study we therefore analyzed the effect of positive margins on ipsilateral breast recurrence and how this may relate to nodal status on long-term follow-up. We were able to analyze a cohort of patients operated between 1987 and1992 who did not necessarily undergo re -excision of positive margins because of the belief at the time that radiotherapy wouls sterilize any positive margins left in-situ and local recurrence could be successfully treated by salvage mastectomy without survival disadvantage.

Our aim of this study was to determine any correlation between margin involvement and nodal status on both local recurrence and overall outcome and to assess if this may have an impact on re-excision policy in individual cases. This study has shown that our overall local recurrence rate (13%) is similar to other units practicing breast conservation surgery at the time. Thus in long term randomized studies the local recurrence rate after breast conservation and radiotherapy was 11% in patients treated by wide excision and radiotherapy in the NSABP B06 study at 12 years of follow-up [3]. In his randomized trial of quadrantectomy versus tumourectomy Veronesi demonstrated an 8% and 18% local recurrence rate at 10 years respectively [15]. Local recurrence rates have reduced because of better case selection, attention to margin status and perhaps because of the effects of adjuvant systemic therapy [8].

We also confirm correlation between involved margins and risk of local recurrence. In addition we have shown a relation between margin positivity and worsening prognosis for node negative patients and a similar, but non significant, trend in women with positive nodal disease. Interestingly, our local recurrence rate of 12% with a 10 years follow-up was identical in both node negative and node positive patients who had negative margins. There were fewer recurrences in margin positive patients who were node positive as compared to those with negative nodes – reflecting the greater early mortality in this group and thus not allowing long enough follow-up for full potential of local recurrence to be realized.

This study shows the impact of local recurrence on prognosis. It adds to the increasing body of evidence correlating positive margins with local recurrence and subsequent overall mortality. Although it has been suggested that positive margins may merely represent unfavorable tumor biology [10] it is clear that in there is now an imperative on the operating surgeon to achieve negative margins when performing breast conservation surgery in all cases. The difference in local recurrence in this study between margin negative and positive cases was less in patients with nodal involvement because of their reduced survival. The current improvements in outcome for breast cancer with enhanced long term survival in all groups indicates the necessity of minimizing local recurrence by good case selection and surgical technique that ensures complete excision of all tumors.

## Conclusion

In conclusion, current knowledge regarding the impact of local recurrence on prognosis and overall improved survival of all patients indicate need for routine re-excision or mastectomy since positive margins are associated with a significantly higher local recurrence rate and reduced overall survival. This is particularly the case for patients with negative nodes and good prognosis. In node positive patients, although we would always recommend re-excision of involved margins, the prognostic value of margins status seems to be of less importance in determining the outcome compared to nodal status, as dissemination is more likely to occur.

## List of abbreviations

LR: Local recurrence; WLE: Wide local excision

## Competing interests

The authors declare that they have no competing interests.

## Authors' contributions

IBC, as first author, has made substantial contributions to acquisition of data, thier analysis and interpretation and has written the background, methods, results and part of the discussion.

MJG has been involved in revising critically for important intellectual content, has given final approval of the version to be published and has written part of the discussion and conclusions.

All authors read and approved the final manuscript.
